# Characteristics of high-dose benzodiazepine use: nationwide cohort study on new benzodiazepine users with 5-year follow-up

**DOI:** 10.1192/bjo.2024.780

**Published:** 2024-09-23

**Authors:** Hanna Särkilä, Heidi Taipale, Antti Tanskanen, Terhi Kurko, Tero Taiminen, Jari Tiihonen, Reijo Sund, Leena Saastamoinen, Jarmo Hietala, Solja Niemelä

**Affiliations:** Department of Psychiatry, Clinical Institute, University of Turku, Turku, Finland; Department of Psychiatry, Turku University Hospital, Turku, Finland; Department of Forensic Psychiatry, University of Eastern Finland, Niuvanniemi Hospital, Kuopio, Finland; Department of Clinical Neuroscience, Karolinska Institutet, Stockholm, Sweden; Research Unit, The Social Insurance Institution, Helsinki, Finland; Center for Psychiatry Research, Stockholm City Council, Stockholm, Sweden; School of Pharmacy, University of Eastern Finland, Kuopio, Finland; Information and Development Services, Finnish Medicines Agency, Helsinki, Finland

**Keywords:** Benzodiazepines, Z-drugs, sociodemographic factors, comorbidity, high-dose use

## Abstract

**Background:**

A nationwide register-based cohort study from Finland including 48 124 incident benzodiazepines and related drug (BZDR) users aged 18–65 years who initiated use in 2006 and were not dispensed BZDRs during 2004–2005. The follow-up was 5 years or until death, whichever occurred first.

**Aims:**

To investigate sociodemographic and clinical factors associated with high-dose use of BZDRs (i.e. Z-drugs) among new BZDR users.

**Method:**

The temporal BZDR dose was calculated as a point estimate every 6 months after initiation as defined daily doses (DDDs) per day, based on the PRE2DUP method (an approach based on mathematical modelling of personal drug purchasing behaviours). Sociodemographic and clinical factors associated with dose categories were studied using multinomial logistic regression.

**Results:**

During the 5-year follow-up, very high-dose BZDR use was observed in 7.4% (*n* = 3557) and medium high-dose use in 25.5% (*n* = 12 266) of the users (corresponding to ≥30 mg and 10–29 mg in diazepam equivalents, respectively). Very high-dose use was more common among men compared with women (10.9% versus 4.6%). Very high-dose use patterns were especially observed in younger age groups (18- to 25-year-olds). Compared with oxazepam, initiating BZDR use with clonazepam (adjusted odds ratio 3.86, 95% CI 3.24–4.60), diazepam (2.05, 1.78–2.36) or alprazolam (1.76, 1.52–2.03) was associated with increased odds for very high-dose use. Both medium high-dose and very high-dose BZDR use were associated with a lower level of education. In all, 58% of very high-dose use occurred in BZDR users who received their first prescription from general practitioners.

**Conclusions:**

Clinicians should be aware of the dose escalation risk especially when prescribing diazepam, alprazolam or clonazepam for psychiatric indications. If BZDRs are needed, our findings suggest favouring oxazepam.

Benzodiazepines and related drugs (BZDRs) have been used since the 1960s,^1,2^ and their use is common in many countries.^3,4^ BZDRs enhance inhibitory gamma-aminobutyric acids (GABA) actions in the brain by allosteric modulation of the GABA-A receptor, and induce anxiolytic, sedating and muscle-relaxing effects as well as anticonvulsive effects. When introduced, BZDRs appeared to be less likely to cause adverse effects, but continuous use of BZDRs may lead into unwanted consequences, such as tolerance, dose escalation, use disorder, respiratory failure and problems in everyday functioning.^5–7^ Even though previous studies on the potential benefits and harms of BZDR use are partly controversial,^8^ many recommendations on the subject have been published over the years.^9,10^ A majority of them emphasise that the dose and duration of BZDR use should be kept as low and short as possible.^11^

Despite the common use of BZDRs, we have very little high-quality knowledge of risk factors for BZDR dose escalation because the definitions of high-dose use differ and real-world data are not easily available in many countries.^12^ Many factors, such as young age, male gender, lower educational level, smoking and alcohol and other substance misuse have been associated with unrecommended high-dose BZDR use.^4,13,14^ However, less is known about factors associated with BZDR dose escalation.^15^

As BZDRs have many known neurobiological and pharmacologic properties that may potentially lead to misuse and physical dependence especially in high doses, the need for further studies is obvious to prevent BZDR-related adverse effects such as cognitive impairment and elevated risks for accidents (vehicle accidents, falls and other traumas). In high doses, BZDRs also pose a risk for toxicity, i.e. impaired coordination, anterograde amnesia, disinhibition, ataxia, vertigo, delirium and inattentiveness, and may lead to fatal overdoses especially when combined with other substances/medications.^2^ With this nationwide register-based follow-up study, our aim was thus to investigate the incidence of high-dose BZDR use as well as the sociodemographic and clinical characteristics associated with subsequent development of high-dose BZDR use, and in particular among new BZDR users. BZDRs are still widely used, and the starting point of the large observational study was to produce further information for clinicians for rational BZDR prescribing.

## Method

**Fig. 1:**
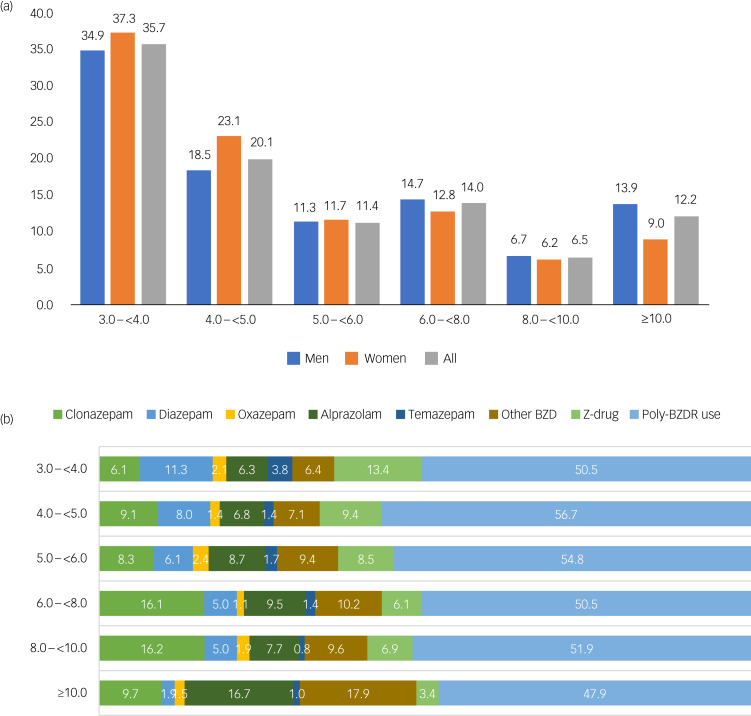
The highest measured dose in defined daily doses per day at any point during the 5-year follow-up period among those defined as high-dose users: (a) distribution by gender and (b) distribution of specific drugs used when the highest dose was observed by very high-dose categories. BZD, benzodiazepine; BZDR, benzodiazepines and related drug.

The study population was collected from the Dispensations reimbursable under the National Health Insurance Scheme register, which is maintained by the Social Insurance Institution of Finland, and contains information on all reimbursed prescription drug purchases. The inclusion criteria for the present study were: (a) initiation of BZDRs during the year 2006, (b) no previous BZDR use from January 2004 until December 2005, and (c) age between 18 to 65 years at initiation. Those who initiated the use with clonazepam or clobazam for epilepsy indication were not included in this study. Those who did not continue BZDR use after the first purchase were excluded, as the dose could not be calculated for these persons. Thus, the final study sample included 48 124 individuals. The follow-up period of this sample was 5 years from the first BZDR purchase. Extremely high BZDR use was also examined in more detail in the >3–≥10 defined daily doses (DDDs)/day groups, as seen in Fig. 1.

This study was based solely on register data and, according to Finnish legislation, no ethics approval or patient consent were needed. The register maintainers pseudonymised the register data before granting access to the researchers, and the study subjects were not contacted. The Strengthening the Reporting of Observational Studies in Epidemiology (STROBE) reporting guidelines were followed.

### Outcome

For defining BZDR use from the Dispensations reimbursable under the National Health Insurance Scheme register, we used the Anatomical Therapeutic Chemical (ATC) classification system, in which the active substances are classified in a hierarchy with five different levels, and further divided into smaller groups based on their qualities and features.^16^

In this study, ATC levels and subgroups used were N03AE01, N05BA (excluding clobazam), N05CD, N05CF and N06CA01 (amitriptyline combined with chlordiazepoxide). Only orally administered dosage forms were included, except for oral suspensions (mainly used for epilepsy indication). BZDRs used in this study were diazepam, oxazepam, alprazolam, zopiclone, zolpidem, temazepam, clonazepam and polypharmacy (two or more drugs concomitantly). Because of the low number of users, the rest of the BZDRs were grouped as ‘all others’, including chlordiazepoxide, lorazepam, clobazam, nitrazepam and amitriptyline combined with chlordiazepoxide.

We used the PRE2DUP method (an approach based on mathematical modelling of personal drug purchasing behaviours)^17^ to calculate the duration of BZDR use, i.e. when the use started and ended. For continuous BZDR use periods, we further calculated a temporal point estimate of dose every 6 months after initiation of use, in defined daily doses (DDDs, i.e. the assumed average maintenance dose per day for a drug used for its main indication in adults), summed from all concomitant specific BZDRs.^18^ This method also ensured that the frequency of purchases or survival bias (e.g. being alive for less than 1 year would skew the results when annually defined cumulative amounts are used) did not affect the result. The DDD reference value for clonazepam is for epilepsy indication (8 mg) and, thus, it was changed to 1 mg and was included only when purchased without special reimbursement for epilepsy (used for other than epileptic indications).

Here, because of the lack of the exact definition for high-dose BZDR use,^3,12,19^ we used a three-class variable with (a) low dose (<1.0 DDDs/day), (b) medium high dose (1.0–<3.0 DDDs/day) and (c) very high dose (≥3.0 DDDs/day). Thus, the utilised measure for high dose, i.e. 1000 DDDs dispensed during 365 days, was reversed to ≥3 DDDs/day (approximating the same amount in daily dose, as 1000 DDDs/365 days = 2.7 DDDs per day, which was rounded up to the nearest whole number). In diazepam equivalent doses, 10 mg of diazepam converts to 1 DDD. The approximate equivalent doses to 10 mg diazepam are given in Supplementary Table 1 available at https://doi.org/10.1192/bjo.2024.780, and are based on the research of Professor Ashton.^20^

The dose category defined for each person was the highest dose that person used in any of the ten time points, measured at 6-month intervals during the 5-year follow-up. For the extremely high BZDR doses, we used categories >3–≥10 DDDs/day.

### Sociodemographic variables

For sociodemographic information, register data from the Finnish Centre for Pensions, Social Insurance Institution of Finland and Statistics Finland were used. The study included users aged 18–65 years, with age categories 18–25, 26–35, 36–45, 46–55 and 56–65 years.

Information on social benefits was used to indicate varying life circumstances at the baseline. The Finnish social security system includes various forms of financial support and benefits, which are meant to ensure that everyone has equal opportunities in working life and society. These data were based on registers maintained by the Social Insurance Institution of Finland and defined as receiving social assistance, labour market subsidy, basic unemployment allowance, national pension, study grants, maternity allowance, paternity allowance, parental allowance and child home care allowance during the year 2005. The Finnish Centre for Pensions provided information on disability pensions, which was measured at the time of BZDR initiation. Information on social benefits was pooled and used as a two-class variable (no/yes) in the multivariate analyses.

Educational level was defined as the highest level the person had completed by the year 2005 and was categorised into three groups: primary and lower secondary education (primary school or less), general upper secondary education/vocational education and training (high school or technical school) and higher education (university). Categories for occupations were defined by utilising the Classification of Occupations from Statistics Finland,^21^ where occupational social class is used as a proxy for socioeconomic position. Data were divided to six bigger categories: ‘managerial/professional’ as groups 1–3 (managers, professionals, technicians and associate professionals), ‘office worker’ as group 4 (clerical support workers), ‘farming/forestry’ as group 6 (skilled agricultural, forestry and fishery workers), ‘sales/industry/cleaning’ as groups 5, 7, 8, 9 (service and sales workers, craft and related trades workers, plant and machine operators and assemblers, and elementary occupations) and ‘unknown’ as group X (unknown).

### Psychiatric disorders

The cohort's potential psychiatric disorders including substance use disorder (SUD) were collected from the Care Register for the Health Care maintained by the Finnish Institution for Health and Welfare, diagnosis recorded in the Disability Pension register, and the Special Reimbursement register maintained by the Social Insurance Institution of Finland. The Special Reimbursement register offered information on people who were granted a special refund for drugs due to certain diagnosed chronic diseases. The information on the special refunds was used as a proxy for the condition. The data were then gathered, utilising the International Classification of Diseases (ICD-10) diagnoses, the Anatomical Therapeutic Chemical (ATC)-codes and special reimbursement codes.

### Statistical methods

The data were analysed using the IBM SPSS software version 27. Dose groups were compared using the *χ*^2^ test for categorical variables and *t*-tests for continuous variables. Multinomial logistic regression was used to estimate odds ratios for medium high-dose and very high-dose users compared with low-dose users. The results are reported as unadjusted and adjusted odds ratios with 95% CI. Data management was conducted with statistical analysis software (SAS) 9.4. Gender, age, level of education, psychiatric disorders including SUD, and social benefits and disability pension status were used as covariates.

## Results

### Sociodemographic characteristics

**Fig. 2:**
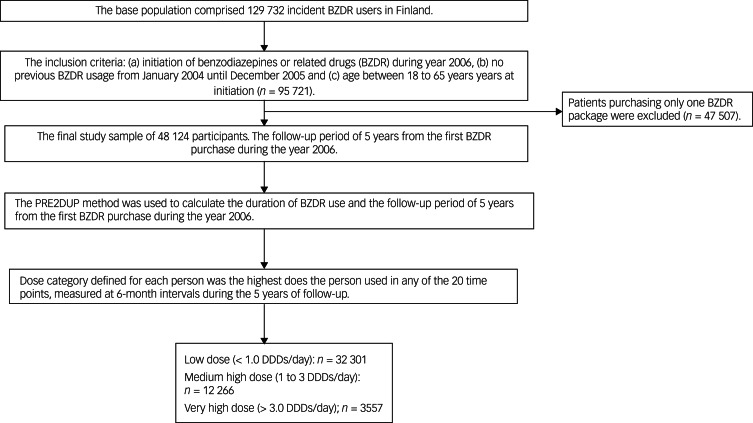
Cohort criteria. DDDs, defined daily doses.

The study cohort included 48 124 individuals (44.4% male, mean age 45.8, s.d. 12.2). The formation of the study sample is presented in more detail in the flowchart in Fig. 2. During the 5-year follow-up, 25.5% (*n* = 12 266) of the study population were defined as medium high-dose users, i.e. their highest measured dose at any time point during the follow-up was 1.0–<3.0 DDDs/day. Very high-dose BZDR use (≥3 DDDs/day) occurred in 7.4% (*n* = 3557) of the study population. During the 5-year follow-up, 12.9% (*n* = 460) of those with very high-dose use had a register-based diagnosis of benzodiazepine use disorder, i.e. ICD-10 diagnosis F13. The respective prevalence was 0.2% (*n* = 80) among the low-dose-users and 1.4% (*n* = 167) among the medium high-dose users. Supplementary Table 4 shows the timing of dose escalation more precisely, in every 0.5 years up to 5 years.

Very high-dose use was more common among men compared with women (10.9% versus 4.6%, *P* < 0.001). Use of less than 0.5 DDDs/day was more common among women, while very high-dose use of ≥3.5 DDDs/day (based on the highest measured dose in any point of the follow-up period) was clearly more common among men compared with women (8.6% versus 3.6%). In the men's group, the dose was significantly higher (mean 1.24, s.d. 1.43) compared with the women's group (mean 0.83, s.d. 1.05).

The distribution of BZDR use in extremely high doses by gender and age is presented more closely in Fig. 1a. Associations between the first dispensed BZDR and BZDR combinations in extremely high-dose BZDR use are presented in Fig. 1b and in Table 1. Initiating with two or more BZDRs, i.e. polypharmacy, was common in extremely high-dose BZDR use. Extremely high-dose BZDR use was clearly associated with initiating with clonazepam and alprazolam, and the most common combinations of BZDRs in extremely high-dose use were diazepam-temazepam and diazepam-zopiclone.

**Table 1:** Highest measured dose in defined daily doses per day and its associations to first dispensed benzodiazepines and related drugs

	*n*	%
Highest dose, anatomical therapeutic chemical classification system		
Diazepam N05BA01	292	8.2
Oxazepam N05BA04	69	1.9
Alprazolam N05BA12	338	9.5
Zopiclone N05CF01	177	5.0
Zolpidem N05CF02	77	2.2
Temazepam N05CD07	85	2.4
Clonazepam N03AE01	372	10.5
Polypharmacy	2030	57.1
Most common two-drug combinations		
Diazepam and Temazepam N05BA01, N05CD07	246	6.9
Diazepam and Zopiclone N05BA01, N05CF01	130	3.7

The associations between sociodemographic factors and BZDR dose group adjusted with gender, age, level of education, psychiatric disorders including SUD, and social benefits and disability pension status are presented in Table 2. The mean age in the very high-dose user group was 36.9 (s.d. 12.76), in the medium high-dose user group 46.4 (s.d. 11.93) and in low-dose user group 47.3 (s.d. 11.65). Very high-dose use manifested clearly in younger age groups. Of the 18- to 25-year-olds, 25.4% had very high-dose use at some point of the 5-year follow-up.

**Table 2:** Associations between sociodemographic background, psychiatric morbidity and subsequent BZDR dose in a 5-year follow-up results of multinomial logistic regression analyses

	Low dose (<1.0 DDDs/day) *n* = 32 301		Medium high dose (1 to 3 DDDs/day) *n* = 12 266		Medium high dose versus low dose	Very high dose (≥3.0 DDDs/day) *n* = 3557		Very high dose versus low dose
	*n*	%	*n*	%	Adjusted^a^ odds ratio (95% CI)	*n*	%	Adjusted^a^ odds ratio (95% CI)
Gender								
Male	12 735	39.4	6311	51.5	1.47 (1.41–1.54)	2321	65.3	2.20 (2.04–2.38)
Female	19 566	60.6	5955	48.5	1.00/reference	1236	34.7	1.00/reference
Age category								
18–25	2026	6.3	904	7.4	1.13 (1.02–1.24)	905	25.4	8.32 (7.15–9.68)
26–35	4254	13.2	1737	14.2	1.24 (1.15–1.34)	873	24.5	5.40 (4.68–6.23)
36–45	7082	21.9	2653	21.6	1.17 (1.09–1.24)	766	21.5	3.19 (2.77–3.67)
46–55	9801	30.5	3743	30.5	1.14 (1.08–1.21)	668	18.8	1.96 (1.70–2.25)
56–65	9138	28.3	3229	26.3	1.00/reference	345	9.7	1.00/reference
Education								
Primary school or less	8743	27.1	4446	36.2	1.00/reference	1842	51.8	1.00/reference
High school/technical school	18195	56.3	6565	53.5	0.79 (0.76–0.83)	1526	42.9	0.53 (0.48–0.57)
University	5363	16.6	1255	10.2	0.61 (0.56–0.65)	189	5.3	0.32 (0.27–0.38)
Occupation								
Managerial/professional	8761	27.1	2063	16.8	1.00/reference	337	9.5	1.00/reference
office worker	2029	6.3	521	4.2	1.09 (0.98–1.21)	86	2.4	1.10 (0.87–1.40)
Farming/forestry	460	1.4	144	1.2	1.33 (1.10–1.61)	18	0.5	1.01 (0.63–1.65)
Sales/industry/cleaning/other	8337	25.8	2770	22.6	1.41 (1.32–1.51)	675	19.0	2.11 (1.84–2.41)
Unknown	12 714	39.4	6768	55.2	2.26 (2.14–2.39)	2441	68.6	4.99 (4.44–5.61)
Social benefits^b^	8842	27.4	5281	43.1	1.49 (1.42–1.57)	2233	62.8	1.91 (1.76–2.08)
Disability pension	4358	13.5	2690	21.9	1.50 (1.42–1.57)	565	15.9	1.65 (1.48–1.84)
Psychiatric comorbidity								
Schizophrenia	1324	4.1	1099	9.0	1.48 (1.35–1.62)	349	9.8	1.05 (0.92–1.21)
Bipolar disorder	486	1.5	358	2.9	1.35 (1.17–1.56)	130	3.7	1.33 (1.07–1.65)
Depression	2323	7.2	1372	11.2	1.24 (1.15–1.33)	390	11.0	0.97 (0.88–1.13)
Attention-deficit hyperactivity disorder	63	0.2	48	0.4	1.36 (0.92–2.02)	29	0.8	1.17 (0.71–1.93)
Substance use disorder	934	2.9	1040	8.5	1.81 (1.64–1.99)	639	18.0	3.20 (2.84–3.62)

BZDR, benzodiazepines and related drugs; DDDs, defined daily doses.

a. Gender, age, level of education, psychiatric disorders including substance use disorder, and social benefits and disability pension status were used as covariates. *n* = 48 124.

b. Receipt of social benefits, including basic social assistance, labour market subsidy, basic unemployment allowance, national pension and study grants, maternity allowance, paternity allowance, parental allowance and child home care allowance during the year 2005.

Both medium high-dose and very high-dose BZDR use were associated with a lower level of education. This was evident for the very high-dose users in particular: 51.8% had completed only primary and lower secondary education. The respective figures for medium high-dose users and low-dose users were 36.2 and 27.1%. As for the occupational information, the number of persons with unknown profession information was notable. Compared with low-dose BZDR use, social benefits and being on disability pension were more common among medium high-dose and very high-dose BZDR users. In total, 62.8% of the very high-dose users received social benefits, and 15.8% were on disability pension.

As seen in Table 2, the psychiatric conditions most commonly associated with very high-dose BZDR use were SUD, depression and schizophrenia. Compared with low-dose use, the risk for very high-dose use was elevated also among BZDR users who were diagnosed with either bipolar disorder or attention-deficit hyperactivity disorder.

Antidepressant and BZDR combinations are presented in Supplementary Table 3. The prevalence of antidepressant use was 22.9% among low-dose users, 30.0% medium high-dose users and 34.6% among very high-dose users, medium high-dose versus low-dose odds ratio (95% CI) 1.44 (1.38–1.51) and very high-dose versus low-dose odds ratio (95% CI) 1.78 (1.65–1.91).

All SUDs were associated with an increased risk for both medium high-dose and very high-dose use of BZDRs, the associations being more pronounced for very high-dose BZDR use. Compared with low-dose BZDR use, very high-dose use was more prevalent among incident BZDR users with a polysubstance use disorder diagnosis (ICD-10 code F19) (0.2% versus 6.8%; odds ratio = 30.78, 95% CI = 23.32–40.63; data not shown in tables). Previous diagnosis of substance misuse was defined as ICD-10 diagnoses from F10 to F19 and are seen in more detail in Supplementary Table 2.

Associations between BZDR use and the first BZDR prescriber are presented in Table 3. BZDRs were mostly initiated by general medicine physicians or those with no specialty, with the total of 26 001 (54.0% of) cases. However, compared with general medicine/no specialty practitioners, the risk for very high-dose BZDR use was the highest among those with a psychiatrist as the first prescriber, and the risk decreased if the prescriber was a neurologist or occupational medicine physician. In the medium high-dose user group, however, the prescriber was most commonly either a psychiatrist or a neurologist.

**Table 3:** Associations between the incident prescribers’ specialty and subsequent BZDR dose – results of multinomial logistic regression analyses

	Low dose (<1.0 DDDs/day) *n* = 32 301		Medium high dose (1 to 3 DDDs/day) *n* = 12 266		Medium high dose versus low dose	Very high dose (≥3.0 DDDs/day) *n* = 3557		Very high dose versus low dose
Prescriber's specialty	*n*	%	*n*	%	Adjusted^a^ odds ratio (95% CI)	*n*	%	Adjusted^a^ odds ratio (95% CI)
Neurology	823	63.4	419	32.3	1.35 (1.20–1.53)	57	4.4	0.59 (0.45–0.77)
Psychiatry	3642	57.0	2057	32.2	1.50 (1.41–1.60)	687	10.8	1.60 (1.46–1.76)
Occupational medicine	4146	77.0	1023	19.0	0.66 (0.61–0.71)	218	4.0	0.45 (0.39–0.52)
General medicine/No specialty	17 406	66.9	6543	25.2	1.00/reference	2052	7.9	1.00/reference
Other specialty	6284	69.4	2224	24.6	0.94 (0.89–1.00)	543	6.0	0.73 (0.66–0.81)

BZDR, benzodiazepines and related drugs; DDDs, defined daily doses.

a. Gender, age, level of education, psychiatric comorbidity including substance use disorder, and social benefits and disability pension status were used as covariates. *n* = 48 124.

Table 4 presents the associations between the active BZDR substance and subsequent BZDR dose. In all dose groups, the BZDRs most commonly purchased first were zopiclone, followed by zolpidem and oxazepam in the low-dose and medium high-dose groups. Among those who initiated with polypharmacy, i.e. initiated with more than one BZDR, 23.4% used BZDR in very high doses at some point in the 5-year follow-up period. With clonazepam initiation, 17.9% of the overall use was in the very high-dose group. With diazepam and alprazolam initiations, the figures were 13.6 and 12.5%, respectively.

**Table 4:** Associations between first dispensed benzodiazepine and subsequent BZDR dose. Results of multinomial logistic regression analyses

	Low dose (<1.0 DDDs/day) *n* = 32 301		Medium high dose (1 to 3 DDDs/day) *n* = 12 266		Medium high dose versus low dose		Very high dose (≥3.0 DDDs/day) *n* = 3557		Very high dose versus low dose	
First dispensed BZDR	*n*	%	*n*	%	Odds ratio (95% C I)	Adjusted^a^ odds ratio (95% CI)	*n*	%	Odds ratio (95% CI)	Adjusted^a^ odds ratio (95% CI)
Oxazepam	5365	73.3	1463	20.0	reference	reference	495	6.8	reference	reference
Clonazepam	805	47.5	588	34.7	2.68 (2.37–3.02)	2.69 (2.38–3.04)	303	17.9	4.08 (3.47–4.79)	3.86 (3.24–4.60)
Diazepam	2158	55.0	1233	31.4	2.10 (1.91–2.29)	1.83 (1.67–2.01)	533	13.6	2.68 (2.35–3.06)	2.05 (1.78–2.36)
Temazepam	1387	54.6	961	37.8	2.54 (2.30–2.81)	2.54 (2.29–2.82)	193	7.6	1.51 (1.26–1.80)	2.05 (1.70–2.47)
Alprazolam	2539	66.9	783	20.6	1.13 (1.03–1.25)	1.27 (1.15–1.41)	474	12.5	2.02 (1.77–2.32)	1.76 (1.52–2.03)
Zopiclone	10 953	70.7	3889	25.1	1.30 (1.22–1.39)	1.50 (1.39–1.61)	656	4.2	0.65 (0.58–0.73)	0.89 (0.78–1.01)
Zolpidem	6970	76.9	1745	19.3	0.92 (0.85–0.99)	1.16 (1.07–1.26)	344	3.8	0.54 (0.46–0.62)	0.78 (0.67–0.90)
All others	1549	52.8	1144	39.0	2.70 (2.46–2.98)	2.69 (2.43–2.96)	243	8.3	1.70 (1.44–2.00)	1.68 (1.41–2.00)
Polypharmacy	575	42.6	460	34.0	2.93 (2.56-3.36)	2.80 (2.44–3.22)	316	23.4	5.96 (5.05–7.03)	5.11 (4.27–6.12)

BZDR, benzodiazepines and related drugs; DDDs, defined daily doses.

a. Gender, age, level of education, psychiatric comorbidity including substance use disorder, and social benefits and disability pension status were used as covariates. *n* = 48 124.

Compared with oxazepam, initiating BZDR use with clonazepam, diazepam or alprazolam was associated with an increased risk for very high-dose BZDR use. This was evident also after adjustments for gender, age, level of education, psychiatric disorders including SUD, and social benefits and disability pension status.

## Discussion

The harmful consequences and adverse effects of inadequate high-dose BZDR use, such as cognitive and memory impairment, increased risk of trauma and other accidents, and the risk of tolerance and use disorder, as well as lower quality of life, have been shown in many studies.^22^ Our findings, utilising national data on all reimbursed prescription drug purchases in Finland, indicate that a high proportion, up to 10% of young men using BZDRs end up using them in very high doses periodically, i.e. ≥3.0 DDDs/day. Initiating BZDR use with clonazepam, diazepam or alprazolam associates with an increased risk for very high-dose use, compared with initiating BZDR use with oxazepam. In all, 58% of very high-dose use occurred in BZDR users who received their first prescription from general practitioners. These findings underline that careful consideration should be taken when initiating these medications.

### Risk for BZDR dose escalation: psychiatric disorders and clinical factors

When initiating BZDRs, certain risk factors should be evaluated. Our results emphasise previous findings^6^ suggesting that especially being male and young age, having a lower level of education and psychiatric disorders including SUD, associate with an increased risk for very high-dose BZDR use, although using BZDRs in general seems to be common in all age groups and genders.^3,22,23^

We found that bipolar disorder and SUD were most associated with very high-dose use of BZDRs compared with low-dose use. All psychiatric conditions except ADHD were associated with very high-dose use. Although BZDRs have their place in the short-term management of, for example, anxiety, psychotic symptoms and alcohol withdrawal, the available evidence does not support long-term or high-dose BZDR use.^24,25^ On the contrary, very high-dose BZDR use has been associated with, for example, neurological and systemic adverse effects in people with schizophrenia^26^ and also adverse effects in treating depression^27^ and anxiety.^28^ In addition, although some previous studies suggest that benzodiazepine misuse is not exclusive to SUD populations,^29^ we found that all specific SUDs were associated with an increased risk for both medium high-dose and very high-dose use of BZDRs.

Our study shows that the largest group, 58% of very high-dose use, occurred with BZDR users who received their first prescription from general practitioners. In addition, compared with other specialties, psychiatrists seemed to be more likely to initiate BZDR treatment resulting in very high-dose BZDR use. As many psychiatric conditions have been traditionally treated with BZDRs,^30^ this seems logical. Acknowledging this, paying attention to current guidelines^31^ seems extremely important for preventing long-term use and dose escalation in the future.^32^ To help clinicians avoid these unwanted results, guidelines and recommendations, such as ‘Smart to avoid recommendations’ (in Finnish) and ‘Do not do recommendations’ from the National Institute for Health and Care Excellence (NICE), have instructions to not routinely offer benzodiazepines to treat, for example, social anxiety disorder in adults, and have been revised recently.

### Risk for BZDR dose escalation: choice of initial BZDR

According to our findings, initiating BZDR use with oxazepam possesses a lower risk for dose escalation compared with diazepam, alprazolam and clonazepam. Alprazolam and diazepam, which are high-potency and quickly eliminating BZDRs, potentially cause more severe withdrawal syndrome than other BZDRs when trying to give up the medication. Diazepam and alprazolam have known misuse potential, and they are commonly available in illegal markets.^33^ Although clonazepam has a long elimination half-life,^34^ it is a high-potency BZDR like diazepam and alprazolam. In our study, clonazepam is also associated with increased risk for very high-dose use compared with oxazepam. Our findings add to the previous but limited clonazepam studies indicating that also clonazepam users have a high risk of developing long-term BZDR use and dose escalation.^7^ Clonazepam also possesses a misuse risk^35^ and can, alone or in combination with other psychoactive substances, lead to adverse outcomes, such as cognitive impairment and aggravation of many psychiatric disorders.^36^

## Strengths and limitations

The main strength of our study is that it has good generalisability, as it included a large nationwide cohort of adults initiating BZDR use. BZDR prescribing policies (and addictions overall) have varied and spread out in the past years, but the problems related to these medications are still, to a somewhat large extent, the same as in 2006–2011. However, this may affect the data and can be considered as something of a limitation. Our classification of BZDRs included the whole range of benzodiazepine-related substances and correlating factors, which has not been explored previously to this extent. In addition, the information sources and study population as well as the utilised methods (such as PRE2DUP) in our study are quite unique and comprehensive because of the Social Insurance Institution's register data and the advanced Finnish healthcare system.

Using prescription registers as the data source also has its limitations. The actual indications for BZDR use except epilepsy were lacking. When it comes to very high-dose use and specifically inadequate use of BZDRs, medications can also be bought from illegal markets without a doctor's prescription.^4,14,37,38^ Hence, our information on the actual amounts of BZDRs used is not entirely comprehensive. The fact that some smaller packages are not reimbursed also affects adversely the data coverage. The correlation of a medication's actual use and its purchase is also questionable, but as some studies show, this is a minor concern.^39^

Certain diagnoses, especially those related to anxiety and substance use, can be somewhat underreported and underdiagnosed among clinicians and, therefore, these estimates may be misleadingly low as we lacked data from primary care. Clinical experience suggests that these comorbidities are much more common than our study suggests.^40^ The fact that the exact indications of prescribed BZDRs were not available may also cause some inaccuracies.

## Conclusions

Our findings from this nationwide register-linkage study implicate that using benzodiazepines above the recommended dose is alarmingly common among people continuing benzodiazepine use after the first BZDR medication purchase. Although BZDRs have many useful qualities in clinical use, their adverse effects relating to high-dose use are indisputable. Certain subgroups, such as young male individuals with comorbid SUD, should be specially noticed as a potentially risky subgroup. Clinicians should be aware of the risk for dose escalation when initiating BZDR use with diazepam, alprazolam or clonazepam. Our findings also suggest favouring oxazepam over the aforementioned BZDRs.

## Supporting information

Särkilä et al. supplementary material Särkilä et al. supplementary material

## Data Availability

H.S. and H.T. had full access to all of the data in the study and takes responsibility for the integrity of the data and the accuracy of the data analysis. The data that support the findings of this study are available from the Finnish government agencies, but restrictions apply to the availability of these data, which were used under licence for the current study and so are not publicly available.
