# Characteristics of high-dose benzodiazepine use: nationwide cohort study on new benzodiazepine users with 5-year follow-up – ADDENDUM

**DOI:** 10.1192/bjo.2025.10944

**Published:** 2025-12-18

**Authors:** Hanna Särkilä, Heidi Taipale, Antti Tanskanen, Terhi Kurko, Tero Taiminen, Jari Tiihonen, Reijo Sund, Leena Saastamoinen, Jarmo Hietala, Solja Niemelä

The above article was originally published with no supplementary files. The supplementary files have now been published.

## Supporting information

Särkilä et al. supplementary material Särkilä et al. supplementary material
