# Supplementary material for: Characteristics of high-dose benzodiazepine use: nationwide cohort study on new benzodiazepine users with 5-year follow-up
Source: BJPsych Open. 2024 Sep 23;10(5):e158. doi: 10.1192/bjo.2024.780 (PMC11457226; doi:10.1192/bjo.2024.780)
Supplement: Särkilä et al. supplementary material [file S2056472424007804sup001.docx]

**SUPPLEMENT**

The DDD is the assumed average maintenance dose per day for a drug used for its main indication in adults. As such, it doesn’t represent or correlate directly to the recommended dose in clinical use. The Equivalence Table is based on the research of [Professor Ashton](https://www.benzo.org.uk/profash.htm) (28). The approximate equivalent doses to 10mg diazepam are given in **Supplementary table 1.**

In **Table 1** of the study, schizophrenia, bipolar disorder, depression, anxiety disorders, and attention deficit hyperactivity disorder (ADHD) were selected among the psychiatric disorders. The codes used were F20-F29/112 for schizophrenia, F30, F31/112 for bipolar disorder, F32-F33/112 for depression, F40-F43 for anxiety disorders, and F90/N06BA for ADHD. The data were gathered from the National Health Insurance Scheme register, Care Register for the Health Care, and Statistics Finland. Substance use disorder is also seen in **Table 1**.

Previous diagnosis of substance abuse was defined as ICD-10 diagnoses from F10 to F19 and seen in more detail in **Supplementary table 2**. Different substance abuse subgroups were explored by setting up categories for alcohol (F10), cannabis (F12), opioids (F11), stimulants (F14-F15), and other/polysubstance (F13, F16, F18, F19) use. Codes F1X.3 were excluded, as they represent the withdrawal state of certain substance use. Only individuals with no other substance use during the defined two-year period were included in the alcohol group. However, alcohol was a possible additional substance used in the other groups (cannabis, opioids, and stimulants). The abuse of BZDRs was analyzed by setting up two groups: those who had been diagnosed with F13.1, F13.2, or F13.4-F13.9 before the start of follow-up, and those who ended up having these diagnoses during the follow-up.

**Supplementary table 3** shows concomitant medication use, which was defined during 30 days before the initiation of BZDR, defined in groups as antipsychotics, antidepressants, antiepileptics, opioids, non-opioid analgesics i.e., non-steroidal anti-inflammatory drugs NSAIDs and/or paracetamol, and for muscle relaxants.

**Supplementary table 4** shows the timing of dose escalation more precisely, in every 0.5 years up to 5 years.
